# The Emerging Role of STING in Insect Innate Immune Responses and Pathogen Evasion Strategies

**DOI:** 10.3389/fimmu.2022.874605

**Published:** 2022-05-10

**Authors:** Qiuning Liu, Saima Kausar, Yingyu Tang, Wuren Huang, Boping Tang, Muhammad Nadeem Abbas, Lishang Dai

**Affiliations:** ^1^ Jiangsu Key Laboratory for Bioresources of Saline Soils, Jiangsu Synthetic Innovation Center for Coastal Bio-agriculture, Jiangsu Provincial Key Laboratory of Coastal Wetland Bioresources and Environmental Protection, School of Wetlands, Yancheng Teachers University, Yancheng, China; ^2^ School of Pharmaceutical Sciences, Wenzhou Medical University, Wenzhou, China; ^3^ Key Laboratory of Insect Developmental and Evolutionary Biology, Chinese Academy of Sciences (CAS) Center for Excellence in Molecular Plant Sciences, Shanghai Institute of Plant Physiology and Ecology, Chinese Academy of Sciences, Shanghai, China; ^4^ State Key Laboratory of Silkworm Genome Biology, Southwest University, Chongqing, China

**Keywords:** microbial infection, STING, host defense, insect immunity, immune factors

## Abstract

Emerging evidence reveals that the stimulator of the interferon genes (STING) signaling pathway in insects and other animal cells helps them to sense and effectively respond to infection caused by numerous types of microbial pathogens. Recent studies have shown that genomic material from microbial pathogens induces the STING signaling pathway for the production of immune factors to attenuate infection. In contrast, microbial pathogens are equipped with various factors that assist them in evading the STING signaling cascade. Here we discuss the STING signaling pathway different animal groups compared to human and then focus on its crucial biological roles and application in the microbial infection of insects. In addition, we examine the negative and positive modulators of the STING signaling cascade. Finally, we describe the microbial pathogen strategies to evade this signaling cascade for successful invasion.

## Introduction

In many living organisms, cytosolic nucleic acid sensing serves as a critical element of immunity. It is a relatively new field of research that involves pathogen detection and insect immune responses to infection. In mammals, the cyclic GMP-AMP synthase (cGAS)-stimulator of interferon genes (STING) signaling pathway is responsible for detecting cytosolic DNA to trigger an effective innate immune response against pathogen infection (1). In this signaling cascade, the binding of cGAS to cytosolic nucleic acid initiates signaling, which stimulates the production of 2′3′ cGAMP, a second messenger protein, and a strong inducer of STING (2–4). Because the cGAS-STING pathway is driven by the presence of nucleic acid rather than pathogen-specific characteristics, it can be activated even when pathogen-specific attributes are not present (5). For this reason, cGAS or cGAS-like molecules of insects can sense a wide range of nucleic acid, both self-origin and foreign. The diverse biological roles of the cGAS-STING pathway in the innate immunity of mammals are comparatively well understood. In contrast, it is an emerging field of study, with its crucial biological roles in host defense becoming clearer as research progresses. Several examples highlight this signaling pathway’s host protective biological role during pathogen infection (6, 7). Recent investigations on the cGAS-STING pathway have revealed that it evolved from an ancient bacterial anti-phage mechanism (8). Emerging evidence shows that the impairment of this extremely diverse innate immune recognition mechanism can disrupt organismal and cellular homeostasis by driving aberrant immunological responses (1). Thus, the investigation is underway or has been undertaken to determine strategies that allow selective regulation of cGAS-STING signaling activity in distinct infection settings (9, 10).

Immune signaling in insects (e.g., *Drosophila*) and mammals have remarkable similarities. *Drosophila* Toll, for example, acts as a receptor for the cytokine Spaetzle rather than a PRR, and uses a conserved pathway to activate transcription factors of the NF-κB family (11, 12). Some PGRP receptors recognize diaminopimelic acid-type peptidoglycan from Gram-negative bacteria, activating the IMD pathway, which leads to the activation of another NF-κB transcription factor, Relish (13). Recent studies have shown that in addition to remarkable conservation in immune pathways, mammals and insects exhibit divergent anti-viral immunity, notably cGAS-STING signaling pathway. Thus, in recent years, researchers have become interested in evaluating the relevance of this immune signaling pathway (11, 13). Despite the fact that several studies on various insect species have discussed the role of cGAS-STING in defense against various pathogens, the research is still in its early stages. These studies improved our understanding regarding the cGAS-STING in insects, many controversial events in diverse insects species need to be investigated further.

In this review, we especially focus on recent advances and developments in the cGAS-STING pathway of insects in comparison with mammals. Further, we discuss its biological role in the induction of innate immune responses against pathogen infections. We describe molecular mechanisms that drive cGAS-STING signaling pathway activity and briefly discuss the current state of understanding of signaling by the cGAS-STING signaling pathway. Outline the current state of knowledge about the structure and modulation of the cGAS-STING signaling pathway in insects.

## Overview of cGAS-STING

It has recently been shown that cGAS-STING signaling pathways involves in various physiological activities of living organisms and have been suggested to remain conserve throughout the evolutionarily period. Although, the purpose of this review is to discuss the involvement cGAS-STNG pathway in insects. In this we will give a brief over of the cGAS-STING pathway in other animals, which will help to understand the evolution of this signaling cascade.

### Fish cGAS-STING Pathway

cGAS being the conserved molecule, has also been reported in fish and appears to regulate immune functions. In teleosts, the N-terminal region of the cGAS protein is markedly conserved, while the amino acids involved in the DNA-binding surface and cGAMP synthesis in the C-terminal male abnormal 21 are also highly conserved (14, 15).

The function of cGAS against pathogens has poorly been investigated in fish in comparison to mammals. In mammals, injection of genomic DNA of pathogens including viruses, bacteria, and parasites into monocyte-derived cells excites immune signals downstream of cGAS. In fish, for example, infection of cGAS knock-out zebrafish with HSV-1 had no effect on induction of ifnϕ1, isg15, and viperin. Rather, the double-knockdown of two zebrafish DNA sensors, DHX9 and DDX41, virtually completely abolished the activation of the anti-viral genes (ifnϕ1, isg15, and viperin). Therefore, it appears that cGAS is hardly involved in the biological defense of zebrafish, and it was considered that cGAS evolved in the animal kingdom after fish (16). Currently, *in vitro* assays and RP-HPLC measurements of cGAMP synthesis have been used to determine specific activities of cGAS variants. Despite the fact that the zebrafish cGAS homologue has a low amino acid identity to human cGAS (less than 35%), their functions similar to that of human cGAS have been evaluated in various studies (17). For example, Liu et al. (18) found that overexpression of zebrafish cGASa/b in HEK293T cells and zebrafish embryos activated NF-κB and type I IFN pathways in a STING-dependent manner, and that cGASa, but not cGASb, was involved in immunoglobulin Z-mediated mucosal immunity in gill-related lymphoid tissue, implying those differential biological roles between the two DrcGASs (18). Surprisingly, it has also been shown that the ortholog of cGAS in grass carp, cGAS-like by interacting with STING suppress expression of type I IFN gene and act as a negative regulator of IFN based response. In addition, it has been reported that the interaction between MITA-TBK1 complex and cGAS, which could partly hinder the process of phosphorylation mediated by TBK1 (19). It has also been suggested that infection of Japanese medaka with the intracellular bacteria, *E. tarda* promotes increased cGAS gene expression in the intestinal tract (14). Collectively, like mammals, fish cGAS, activates NF-κB, causing STING-mediated generation of type I IFN. It may, on the other hand, be involved in humoral response mediated by immunoglobulin Z, through this has not been proven in mammals. It is still debatable whether fish cGAS is highly effective in preventing pathogen infection.

### Avian cGAS-STING Pathway

In birds, in response to microbial infections, the cGAS-mediated DNA detection and activation leading to 2′3′-cGAMP synthesis has been thoroughly reported. Like other animals, 2′3′cGAMP acts as a ligand to the STING, which dimerizes and oligomerizes upon ligand binding, resulting in its activation and downstream stimulation of type I IFNs, contributing to an antimicrobial state (20, 21).

The avian cGAS, like that of other animals, is triggered by direct binding to DNA in a sequence-independent manner (22). When DNA is bound to cGAS dimers, conformational changes occur, resulting in cGAS enzymatic activity (23). GTP and ATP is used by activated cGAS to synthesize the endogenous second messenger 2′3′cGAMPby (24). STING conformational changes are triggered by 2′3′cGAMP binding, culminating in STING closure confirmation and oligomerization. STING endoplasmic reticulum retention is mediated by interaction with the Ca2+ sensor stromal interaction molecule 1 in the resting state (25). STING traffics through ERGIC and the Golgi apparatus after cGAMP binding-induced conformational changes, where it interacts with TBK1, resulting in direct phosphorylation by TBK1. Finally, the C-terminal tail region of STING serves as a docking site for IRF3, which is subsequently phosphorylated by TBK1 and activated, dimerized, and translocated to the nucleus to regulate the type I IFNs transcription (26). Avian cGAS have a shortened N-termini with the least similarity to human cGAS (27). Unlike goose cGAS, the N-termini of chicken and duck cGAS appeared to be rich in positively charged amino acids, suggesting biological roles related to nuclear localization and DNA binding. For DNA recognition and cGAMP synthesis, the nucleotidyl transferase domain was determined to be both essential and sufficient (22). The Avian NTase domains exhibited a greater level of similarity to human cGAS; however, this level of similarity might vary with different avian species. The catalytic residues (glutamate (Glu) 225, aspartate (Asp) 227, and Asp319 in humans) appear to be conserved in avians that are located on the centrally twisted β-sheets of the α/β core, and catalytic pocket is formed between the α/β core and the helix bundle (22). Furthermore, the activation loop in the vicinity of the catalytic pocket (residues 210–220 in humans) that contributes to nucleotide recognition and is crucial for the catalytic activity of cGAS also appeared to be conserved in avian cGAS. On the opposite side of the catalytic pocket, the long N-terminal helix, and the CCHC-type zinc finger are critical for DNA recognition in human cGAS. Goose cGAS lacked the spine helix, while the spine helix of duck and chicken cGAS has conserved leucine, suggesting that human cGAS and avian have similar mechanisms of dsDNA binding and catalytic site rearrangement. The absence of spine helix in goose cGAS suggests that the N-terminus is involved in dsDNA recognition and conformational activation.

Endoplasmic reticulum adaptor STING binds 2′3′cGAMP to activate downstream innate immune signaling. Avian STING has a low amino acid identity (less than 50%) to human STING. Besides their low similarity, amino acids necessary for the recognition of diverse CDN moieties remained conserved, indicating that CDN recognition and STING activation have similar mechanisms. Notably, C-terminal tails of avian STING contain pLxIS motif similar to that of human STING, suggesting that birds and human STING have conserved actions of TBK1-mediated phosphorylation and binding of IRF7 to promote downstream signaling (28). The characterization of duck STING in BHK21 or DEF cells showed that it is located in the endoplasmic reticulum and mitochondria. Additionally, overexpression of STING resulted in considerable activation of NFκB, IFN-β, and ISRE promoter elements as well as an anti-viral impact against DPV infection (29).

### CGAS-STING Pathway in Other Vertebrates

In vertebrates, the cGAS–STING signaling pathway is highly conserved, with cGAS sharing the ability to bind dsDNA *via* a zinc-ribbon and produce cyclic dinucleotides, such as 2′3′-cGAMP.STING has developed a C-terminal tail since the teleost fish, which allows it to phosphorylate TBK1 and recruit IRF3, signaling the generation of type I IFNs (30). Although, this signaling cascade has been well studied in mammals and many other vertebrates, our understanding is limited regarding the amphibians and reptiles. Although, STING signaling in amphibians has been described, of note, some amphibians, such as *Xenopus tropicalis* and *Xenopus laevis*, appear to have lost the STING CTT domain (27). In X. tropicalis STING has been shown to bind 2′3′-cGAMP (31), but its signaling functions have never been investigated. It is possible that some signaling outputs of STING activation, such as NF-kB activation or autophagy, do not require the CTT. It would be interesting to see if STING signaling is important for IFN synthesis and/or viral control in Xenopus as it is in other vertebrates (16, 16, 32, 33), and whether the other downstream signaling outputs are still achieved in the absence of the CTT.

Reptiles are another major group of animals on the land. Besides their occurrence, the DNA sensing in reptiles is less understood due to a lack of genomic sequencing and immunological research; however, recent sequencing of the wall lizard genome has revealed the absence of TLR9 and the presence of the ortholog of the human multilectin receptor DEC-205 in addition to STING signaling. This receptor has also been reported other vertebrates (34, 35), and has been suggested to be a cell surface receptor for CpG-ODNs (35, 36). These evidences suggest that both the amphibians and reptiles are less studied group of animals with regard to the cGAS-STING signaling. Therefore, extensible research is required to determine the components of STING signaling and how they control various physiological processes in these two groups of animals. Furthermore, such studies will also assist in precisely understanding the evolution of the STING signaling cascade.

### Insects cGAS-STING Pathway

The production of cGAMP in the cytoplasm of a cell is the first and most important step in activating the STING signaling cascade in mammals (2, 3, 37, 38). After interactions with cytosolic double-stranded DNA, the cGAS catalyzes the synthesis of 2′3′-cGAMP, a second messenger molecule in the cGAS-STING signaling cascade (3). Subsequently, by assembling the STING-Tank-binding kinase 1 (TBK1)- interferon regulatory factor 3 (IRF3) signalosome, it activates the STING mediated signaling cascade, resulting in the stimulation of biological effects in response to infection (39).

The cGMP-AMP signaling pathway, which shows striking similarities to their mammalian counterparts, has recently been discovered in a variety of insect species (40, 41). In contrast to other insects, the structural components of this signaling pathway have been well described in a model insect species, *Drosophila* melanogaster (41). *Drosophila* CG1667 was proven to be an ortholog of the mammalian STING protein by Martin et al. (42), who also demonstrated that STING has a conserved architecture that is necessary for it to bind with cyclic dinucleotides (CDNs). Of note, Kranzusch et al. (31) observed that insect STING orthologs, including *Drosophila* STING, did not bind to CDNs in their *in vitro* experiments. In these experiments, they used a full-length *Drosophila* STING protein that included the hydrophobic N-terminal transmembrane domains that may inhibit CDNs binding to the STING protein, particularly when the protein is not precisely folded in its natural state *in vivo*. Crystal structures of *Drosophila* STING may be needed to uncover its ability to precisely bind with CDNs. In contrast to mammals, the cGAS homolog(s) responsible for the sensing and binding with double-stranded DNA and generating second messenger CDNs for STING activation have remained unidentified in the fly. There are several genes in the insect genomes that encode enzymatic proteins that contain a catalytic region named as a nucleotidyltransferase domain, which is structurally and functionally similar to the catalytic domain of cGAS. However, none of these enzymatic proteins contain the DNA-binding motif that is an essential component of cGAS. Recent two articles publications in Nature have shown that the cGAS-like receptors (cGLRs) in the fly can sense nucleic acid and trigger the synthesis of CDNs, which then activates of the STING signaling cascade, which is responsible for inducing immune effector molecules (43, 44). The findings of these two studies contribute to our understanding of the STING signaling cascade in insects.

**Figure 1:**
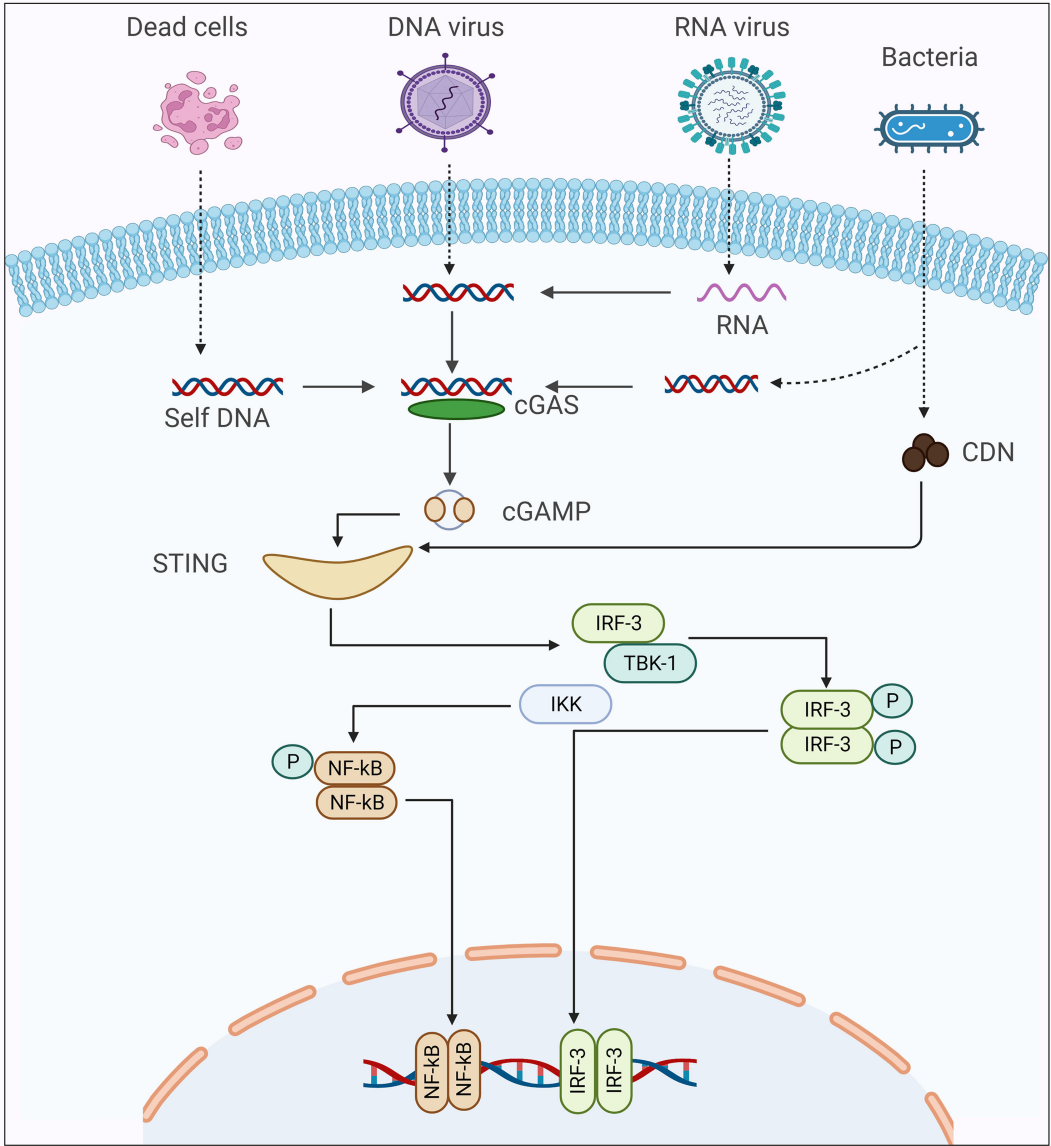
The Schematic presentation of cGAS-STING signaling cascade activation in response to different kinds of pathogens.

Using homologs of *Tribolium castaneum* cGLR, Slavik et al., recently isolated ~53 recombinant cGLR proteins from *Drosophila*, and then biochemically investigated the nucleotidyltransferase enzymatic activity of these proteins (43). Among these proteins, they observed that *Drosophila* cGLR1 has the ability to sense double-stranded RNA and catalyzes the synthesize of second messenger (3′2′-cGAMP), which can activate the STING-signaling cascade in the dipteran insects. Using structural and sequence comparison of *Drosophila* cGLR1 and *Tribolium castaneum* cGLR, the authors identified a conserved architecture, which includes similarities in a nucleotide signaling core and a primary ligand binding surface, which is responsible for detecting double-stranded RNA and for the enzymatic activity of *Drosophila* cGLR1 (43). Concurrently, Holleufer et al., also concluded that *Drosophila* cGLR1 could recognize double-stranded RNA and is responsible for the synthesis of 3′2′-cGAMP, which is the major second messenger that preferentially binds to STING, resulting in the activation of the downstream signaling cascade (44). However, Slavik et al. did not investigate the enzymatic activity of Drosophila cGLR2, and Holleufer et al. did not isolate Drosophila cGLR2 recombinant protein (43, 44). Of note, cGLR1 synthesizes 3′2′-cGAMP rather than 2′3′-cGAMP. The phosphate bond positions of 3′2′–cGAMP are opposite of those of 2′3′-cGAMP. This reversal arises by a switch in the order in which the cGAMP-forming nucleotides interact to the enzymatic protein. In human HEK293T cells, it has been shown that the cGLR2 protein can synthesize both 2′3′-cGAMP and 3′2′-cGAMP to sense genomic material, whereas, in insects, it has been reported that cGLR2 can produce both of these second messengers, but the enzymatic activities have not been confirmed in either *in vivo* or *in vitro* experiments (**Figure 1**) (44). Interestingly, Hua et al., discovered the increase in cGAMP silkworm, *Bombyx mori*, following recognizing double-stranded DNA (40). However, the mechanism of cGAMP production in the silkworm was not reported in this study, it is possible that the cGAS-STING signaling pathway is activated in insects in response to the presence of double-stranded DNA in the cytoplasm of the insect.

Like its mammalian counterpart, the insect STING protein resides in the endoplasmic reticulum, and its activation is required for downstream signaling (41). STING is translocated from the endoplasmic reticulum to the Golgi apparatus in mammals *via* the endoplasmic reticulum-Golgi intermediate compartment (45), but the precise understanding of this mechanism remains incomplete, even in mammals, due to the lack of detailed molecular steps and association between different putative factors involved in this trafficking process. What we know is that the endoplasmic reticulum to apparatus transport machinery, which consists of coatomer protein complex II vesicles and is dependent on the GTPase SAR1A and the COPII complex components, including SEC24C and the ARF-GTPase ARF1, facilitates transport of STING protein. STING protein recruits TANK-binding kinase 1 (TBK1) to initiate downstream signaling after reaching at the golgi compartments (46). STING to IKKb-Relish axis signaling has been demonstrated in insects, such as *Drosophila* by Goto et al. (41), and is described in more detail later in this review.

## STING and Anti-Viral Immunity in Insects

Insects efficiently produce antimicrobial peptides in response to fungal and bacterial infections. TOLL and IMD, two evolutionary conserved immune signaling pathways, govern the production of these peptides: TOLL, IMD, and JAK-STAT, in addition to RNA interference (RNAi), have recently been reported to be implicated in the host defense against certain viruses. Anti-viral immune responses, on the other hand, rely on RNAi to detect and process intracellular viral double-stranded RNAs. In addition, a large number of genes transcription levels are increased due to viral infection, suggesting an induced immune response against viral infection (47). Invertebrates, such as oysters have been shown to have IFN-like immune responses against viral infection, implying that such a response exists in other invertebrates, including insects (48). The biological role of STING in insect innate immunity has recently been come into the spotlight. Insects’ ability to detect and neutralize virus infections is still unknown. When cytosolic nucleic acid ligands sense viral nucleic acid in mammals, they stimulate STING, which then activates type I interferon and NF-kB immune responses, reducing viral infection (33). Insects, like their mammalian counterpart, also express STING orthologs but how they participate in anti-viral immune responses were obscure.

**Figure 2:**
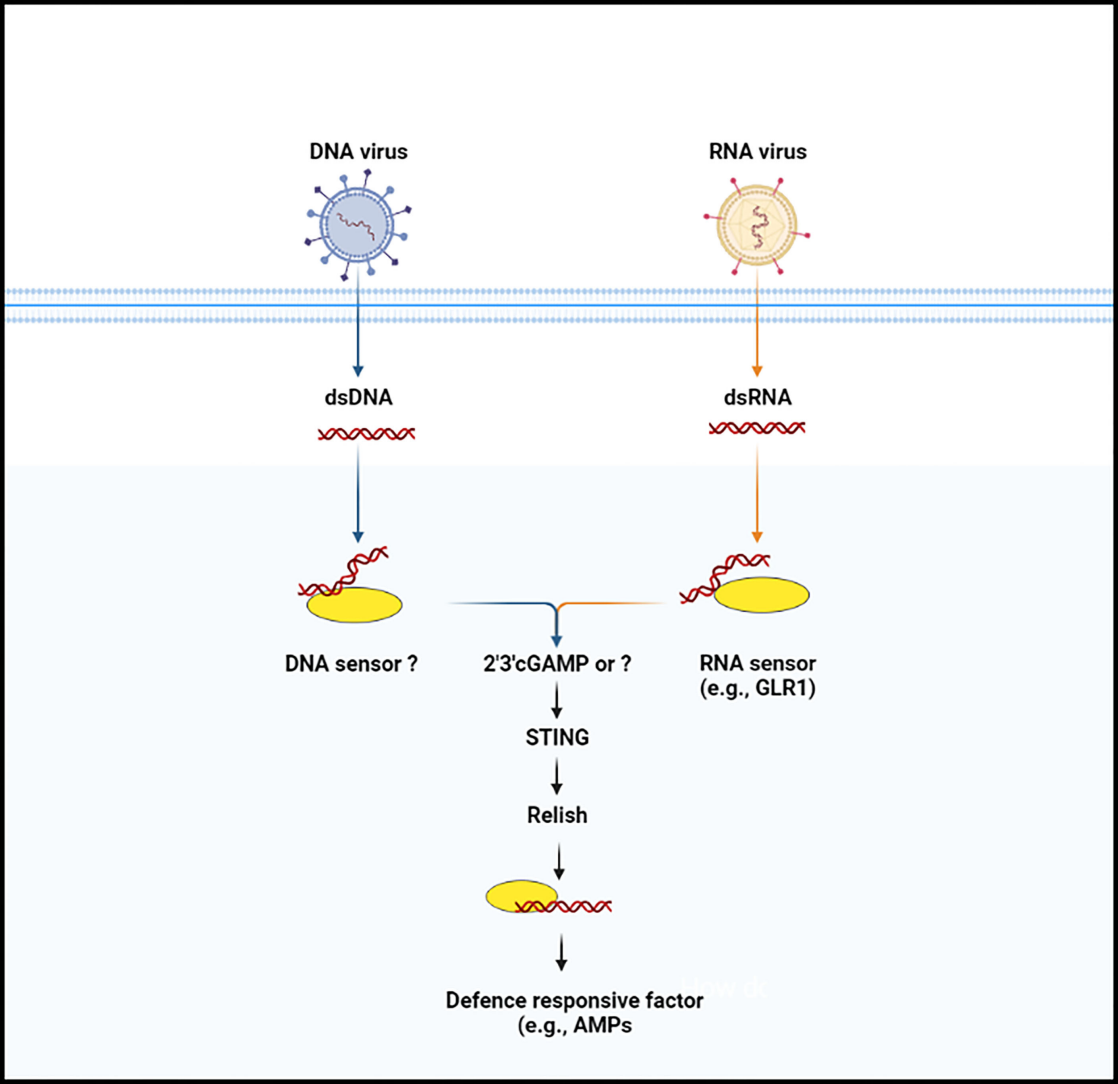
The anti-microbial defense signaling pathway (cGAS-STING pathway) in insects. Nucleic acid (DNA or RNA) is not usually occurred in the cytoplasm of the insect cells. When the enzymatic protein cGAS (cGLR or unknown molecule) recognize nucleic acid, they lead to trigger the synthesize of secondary messenger molecules (e.g., 2’3’-cGAMP). Following the synthesize of secondary molecules, STING protein is induced, which then binds with the Relish and promotes its translocation, by which in induces anti-microbial factors.

STING was discovered by a group of scientists who were investigating the molecular mechanisms underpinnings of the innate immune response against Zika virus. Buchon and co-workers found that viral infection activates the IMD pathway activation in the *Drosophila* brain and that mutant *Drosophila* for Relish or peptidoglycan recognition protein-LC and -LE, two PRRs that induce the IMD pathway, were more susceptible to viral infection than controls. The authors also noted that Zika virus infection-induced STING expression and that this stimulation was dependent on Relish, suggesting that STING has biological roles as an NF-κB-modulated anti-viral effector (49, 50). In addition, Lamiable et al., followed up on the discovery that several DNA viruses of insects independently hijacked a gene encoding an immunoregulatory cytokine, suppressing the activation of the IMD signaling pathway (51). These findings prompted a thorough examination of IMD pathway’s role in anti-viral innate immunity, which showed that two components of the pathway, including Relish and kinase IKKβ, but not the entire pathway, are required to resist infection by two picorna-like viruses *in vivo* and a cell line. Another study performed epistasis analysis and Nazo (an anti-viral factor) expression as a readout to explore the link between STING and the IKKβ- and Relish axis. The authors observed that the over-expression of STING resulted in a considerable increase in Nazo the transcript levels. But when IKKβ was suppressed, Nazo expression was equal to that of the control group, implying that STING is potential regulator of Nazo upstream of IKKβ. Of note, STING over-expression was disrupted by the suppression of Relish, IKKβ, and Nazo (anti-viral factor), implying that this process regulates anti-viral gene expression independently of the IMD pathway (41). Later on, Cai and co-workers injected naturally existing CDNs and noted that four of three CDNs induced the STING-regulated genes, which are important in *Drosophila’s* defense against viral infections. In addition, the authors reported that this protection was entirely based on *Drosophila* STING and Relish and that ATG7 and AGO2 were not required. Although the biological role of most the STING-regulated genes is unknown, Nazo and Vago have been shown to have a role in anti-viral resistance (41, 52, 53). Interestingly, a study on another insect (*Bombyx mori*), using a Liquid Chromatography coupled with tandem Mass Spectrometry analysis, confirmed the production of cGAMP in the cytoplasm of BmE cells. They also discovered a human STING homologous protein in this species, which they linked to Relish. The authors noted that infection with nucleopolyhedrovirus (NPV), (a DNA virus from the Baculoviridae family) stimulates the co-expression of STING and Relish protein, and any change in STING concentration affect the synthesis of Relish protein. After binding with Dredd protein, STING activates Relish and promotes its nuclear translocation. Finally, they proposed a conserved cGAMP–STING–NF-kB signaling axis that protects silkworm from NPV infection by increasing antimicrobial peptides (e.g., gloverin and cecropin) production (**Figure 2**) (40). This study opens up new avenues for research into the anti-viral immune response of insects, and more evidence from other insect species will help to clarify this anti-viral defense mechanism in insects.

Recently, Holleufer et al. (44) used a genetic approach to identify cGAS-like enzymes, including cGLR1 and cGLR2 and concluded that both these proteins respond to viral infection in *Drosophila*. They noted that an equal amount of these enzymes was expressed in flies and cells cultured *in vitro* and that this was enough to induce STING. *Drosophila* with cGLR1 mutations behaved similarly to STING mutants, with a decrease in the activation of various STING-associated genes (Srg1, Srg2, and Srg3), and an increase in infection by Kallithea virus (a DNA virus) and *Drosophila* C virus (an RNA virus) as well as reduced survival. However, the author noted that while cGLR2 mutant alone does not produce the same results as the cGLR1 mutants, the cGLR2 enzyme has some overlapping biological roles with cGLR1, as evidence by the fact that double-mutant *Drosophila* lacking both the cGLR1 and cGLR2 genes is more susceptible to infection by Kallithea viruses or *Drosophila* C virus than a single knockout. In addition, compared to a single knockout showed a higher level of viral replication, a lower survival rate, a more-severe abnormality in the activation of STING-modulated genes, implying that both genes can trigger viral infection signaling and promote fly survival. In addition, natural *Drosophila* pathogens, the authors investigated the effects of vesicular stomatitis virus (RNA virus) and invertebrate iridescent virus 6 (DNA virus), although neither virus caused any overt phenotype for viral load and survival in cGLRs mutants. Furthermore, these viral infections did not result in a robust STING response, implying that 3′2′-cGAMP and STING are suppressed by a viral molecular mechanism that has yet to be discovered.

The second group of researchers identified cGAS in fly using different research techniques and offered a precise mechanism of STING activation and anti-viral immune responses (43). They discovered that gene CG12970 is a *Drosophila* cGLRs that is similar in architectural and biological relevance to cGAS and termed it cGLR1 based on the sequence analysis and systematic biochemical screening. The cGLR1 requires double-stranded RNA ligands that are longer than the 21-23-bp RNA molecules commonly produced during RNA interference in *Drosophila*, indicating that self-recognition is avoided specifically.

Insect cGLRs have a similar ligand detection mechanism to human cGAS, suggesting that insect cGLR1 solely senses a foreign RNA. The authors also reported that *Drosophila* STING has a highly conserved V-shaped homodimeric architecture with a deep central pocket that binds to 3′2′-cGAMP. The STING–3′2′-cGAMP structure has a tightly ‘closed’ conformation with STING protomers, similar to the closed conformation of human STING bound to 2′3′-cGAMP (38), demonstrating that STING activation is driven by specific 3′2′-cGAMP-dependent signaling. Finally, they show that D. *melanogaster*, a cGLR-STING-NF-κB axis protects animals from viral replication by activating the synthesis of the anti-viral genes (STING regulated genes).

Altogether, insect STING modulates NF-κB-dependent immune responses and is involved in anti-viral innate immunity. The discovery of genes encoding cGAMP in the genomes of insect (dipteran, lepidopteran) hosts suggests that cGAS-STING signaling plays an important role in insects anti-viral innate immunity. So far, different anti-viral factors, including antimicrobial peptides, have been reported; however, further evidence is required to fully understand the anti-viral responses induced by cGAS-STING in insects.

## STING-Dependent Autophagy in Insects

Autophagy is an ancient biological process that is involved in a variety of physiological activities, such as immune responses, development, and aging. Autophagy also involves in degrading aggregated or misfolded proteins, eliminating damaged cellular organelles, including endoplasmic reticulum, mitochondria peroxisomes, and also abolishing intracellular microbial pathogens (54). This process starts with various stressors and leads to the targeted isolation of cytoplasmic contents within autophagosomes, which then merge with lysosomes to break down the engulfed cargo. Autophagy has been implicated in the host immunological defense against a variety of intracellular microbial pathogens in insects and other animals. Upon the pathogen infection, innate immune signaling induces autophagy, which degrades intracellular viruses, bacteria, and parasites (55). Thus, autophagy has emerged as a crucial biological process for suppressing pathogen replication or pathogenicity and host survival.

**Figure 3:**
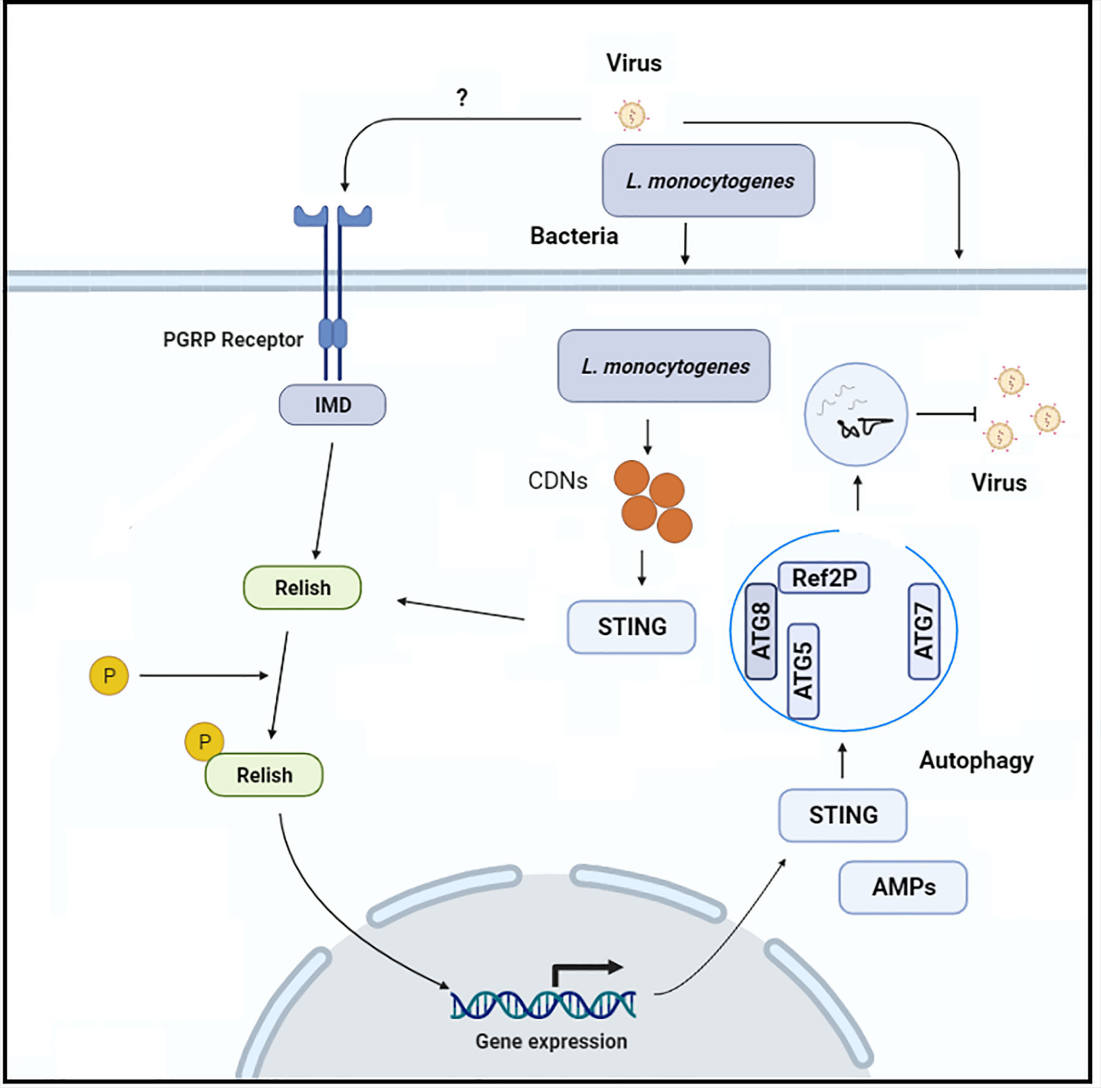
the Schematic representation of the molecular mechanism through which Rel-NF-_K_B-dependent STING expression induces anti-viral autophagy in insects.

Zika virus, an arthropod-borne virus, infection leads to severe complications as neuroprogenitors are lytically infected. Like other arboviruses (e.g., West Nile virus), which infect the central nervous system of mosquitos and are regulated by poorly understood immune mechanisms, Zika virus particularly infects and replicates in adult *Drosophila* brains (56, 57). Liu et al. (55), explored innate immune mechanisms that suppress the Zika virus infection in the fly brain. They discovered that the anti-viral RNA interference pathway is not involved in the suppression of Zika virus replication, implying that the virus encodes an RNA interference silencing suppressor. However, because *Drosophila* mutant for Relish/NF-kB transcription factor had a greater viral infection, the authors concluded that Zika virus infection in the fly brain promotes the Relish/NF-kB/IMD pathway, which is implicated in viral infection suppression. This canonical Releish/NF-kB pathway is stimulated by the pattern recognition receptors, peptidoglycan recognition proteins-LE or peptidoglycan recognition proteins-LC, which interact with bacterial peptidoglycans either intracellularly or extracellularly, respectively (**Figure 3**). The authors observed that flies mutant with both of the above-mentioned receptors are more vulnerable to Zika virus infection. However, the authors were unable to determine whether these receptors bind to the Zika virus directly or indirectly to trigger this protective cascade. In addition, they found a Rel/NF-KB-dependent induction of STING in the fly brain in response to Zika virus infection and the fact that STING is a downstream factor of Rel/NF-KB, implying that STING is involved in the brain’s anti-viral immune response. Because this signaling cascade in insects does not activate IRF3-dependent type I interferons signaling, the authors assumed that STING is likely to govern anti-viral autophagy. They supported this hypothesis by examining autophagy-related factors (Atg5, Atg7, Atg8-II, and mCHERRY-Atg8 puncta), and they concluded that autophagy is a critical process controlling Zika virus replication because they observed an increase in the Zika virus replication after autophagy-related factors were lost (55). The molecular mechanism by which STING activates autophagy, and the viral pathogens cargo target for degradation remains unknown. In contrast, a recent study generated ATG7 mutant *Drosophila* and injected them with *Drosophila* C virus. Interestingly, the authors argue that 2′3′-cGAMP, along with Drosophila STING and Relish, regulate viral infection independently of the canonical autophagy pathway route, resulting in a reduction of viral infection (53). Based on these studies, it appears that the host may have a virus-specific role or that an unconventional autophagy pathway is induced to eliminate a viral infection. However, more research is required to fully understand STING-dependent autophagy in insects. Recently in another study, suggested that silkworm (*Bombyx mori*) innate immune responses (autophagy) against parasite, *Nosema bombycis* infection are STING-dependent comparable to those observed in vertebrates (58). The authors reported that *B. mori* ATG8, an autophagy-related factor, was up-regulated after *N. bombycis* infection. In addition, after *N. bombycis* infection, they noted that the level of LC3 (biochemical hallmark of autophagy) in the midgut of BmSTINGΔ6bp/WT, and BmSTINGΔ5bp/WT (STING knockout transgenic lines) is lower than that of wild type *B. mori* (40), suggesting that loss of STING is linked with LC3. In insects, this change in the level of autophagy-related protein suggests that STING control autophagy LC3 protein. However, in this study, at the latter stage of infections, all of the transgenic lines infected with *N. bombycis* died. This phenomenon raises several questions about the possible causes of larval death during the latter stages of infection, such as whether the host’s resistance mechanism is insufficient to completely remove the parasite or whether the parasite has managed to hijack autophagy and redirect this process to support their replication within the host (59, 60). Hua et al. (40), investigated the possible mechanism of the parasite to take over host immunity. In their study, the authors found that *N. bombycis* infection stimulates silkworm protein degradation, which in turn supports in the synthesis of host ATP, which is necessary for delivering nutrients and energy to invading pathogens (61). A detailed molecular mechanism is needed to explore to understand the resistance mechanism of the host against the parasites. However, this study opens new research avenues to investigate how insects respond to parasitic pathogens. The ERGIC is formed in mammals when STING binds to cGAMP interacts with SEC24C, which causes the endoplasmic reticulum to produce COP-II vesicles, which eventually form the ERGIC. The ERGIC serves as a membrane source for WIPI2 recruitment and LC3 lipidation, which results in the production of autophagosomes that target cytosolic pathogens for degradation by the lysosome. Of note, the stimulation of LC3 in response to cGAMP is the characteristic feature of mammalian STING and is due to the presence of TBK1 activation domain at the C-terminus of the protein. It has been shown that the C-terminal architecture of insects STING, especially the *Bombyx mori* STING, is highly conserved with human and mouse STINGs (40). Thus, it seems that insect STINGs may retain the ability to induce autophagy response as a result of the conserved architecture at the C-terminus. In addition, although STING in some vertebrates, such as sea anemone, lacks the TBK1 activation domain, it is still capable of stimulating LC3 in response to cGAMP stimulation (31). Thus, autophagy stimulation is an ancient and highly conserved biological role of the cGAS-STING signaling pathway in animals (46).

Overall, it seems that STING-dependent autophagy is a conserved physiological process in insects, in addition to the production of anti-pathogen factors to eliminate microbial infection similar to those of vertebrates counterparts (62, 63). However, many questions like most of the STING-dependent autophagy factors and their mechanism of function is unknown. It is also a matter of interest whether induction of autophagy in insect host is a general mechanism against different pathogens, e.g., viruses, or this process only activated against specific viruses. Experimental evidences are also required to ensure whether insect host induces STING-dependent autophagy in addition to the synthesis of antimicrobial factors. Future studies will help to understand these questions.

## Role of STING in Anti-Bacterial and Antiparasitic Immunity in Insects

So far, most of the studies addressed the role of cGAS-STING signaling in the host defense against viruses. In some studies, it has been reported that STING plays a crucial role in the prevention of bacterial infection. Martin and co-workers were the first to report STING mutant *Drosophila* had dysregulation of the IMD pathway, which resulted in reduced resistance by the intracellular bacterium *Listeria monocytogenes.* In this study, the authors demonstrated that CG7194, the closest homolog to cGAS in flies, did not cause any change in mortality against *L. monocytogenes* infection, suggesting that CG7194 (cGAS homolog) is not involved in activating STING signaling in bacterial infection and that signaling could be induced by the bacterial CDN 3′3′c-di-GMP. It appears from these results that in *Drosophila*, STING functions as a direct sensor of bacteria through binding of CDNs and activates an IMD- and Relish-dependent anti-bacterial response (42). They went on to demonstrate that STING can bind to c-di-GMP, and that a mutant protein product lacking a CDN-binding domain can completely abolish this binding. While *Drosophila* STING is structurally varying to mammalian STING, as it lacks the CTT, which is an essential region for monitoring downstream signaling transduction to IRF3. Mutants of mammalian STING that lack CTT are unable to activate IRF3, although this mutant has the ability to activate NF-κB signaling. Likewise, *Drosophila* STING has the ability to stimulate NF-κB signaling. After infection with *L. monocytogenes*, the release of c-di-GMP promotes the *Drosophila* IMD signaling pathway, resulting in increased expression of IMD-related antimicrobial peptides (AttacinA and CecropinA2). It is possible that STING operates through Relish to activate the IMD pathway, as evidenced by the reduction in cleavage and activation of Relish in STING knockout flies. In addition, the authors ruled out the possibility of involvement of the Toll signaling pathway because they did not observe any change in the Toll-related antimicrobial peptides (drosomycin) during the experiment. Infection with *L. monocytogenes*, STING-overexpressing flies have a lower bacterial load, lower mortality, and decreased susceptibility to the infection (42). In addition, the AttacinA and CecropinA2 genes have highly expressed these flies. The depletion of Relish or IMD in Drosophila STING-overexpressing flies results in a reduction in the production of antimicrobial. Interestingly, the *Drosophila* CG7194 putative cGAS homolog lacks a zinc ribbon domain and a positively charged N-terminus, both of which are required for DNA binding (4). The depletion of *Drosophila* CG7194 has no effect on the mortality rate of insects infected with *L. monocytogenes*, indicating that *Drosophila* CG7194 may not be involved in innate immune responses against bacterial pathogens (42). The overall conclusion is that *Drosophila* STING directly senses bacterial CDNs, especially c-di-GMP, and triggers anti-bacterial immunity *via* IMD-Relish signaling in the absence of a functional cGAS ortholog.

In contrast, a previous study reported that the Toll and IMD signaling pathways in *D. melanogaster* were activated in response to the infection with *L. monocytogenes*. The authors demonstrated that *dif* or IMD mutant flies are more susceptible to *L. monocytogenes* infection, suggesting that this bacterial infection can promote the induction of Toll and IMD pathway in the fly (64). Another study conducted with a bacterial pathogen, *L. monocytogenes* in *Tenebrio molitor*, recently saw a similar outcome (significant increase in IMD and Toll specific antimicrobial peptides) with the same conclusion. The authors suggested that, in addition to antimicrobial peptides, Relish regulates the autophagy-related proteins (e.g., serine-threonine protein kinase), since they saw significant downregulation in autophagy-related proteins *T. molitor* that was deficient in Relish expression (65). The induction of IMD, Toll signaling pathways, and autophagy in response to *L. monocytogenes* infection suggest that there may be possible cross-talk between Toll and IMD signaling and autophagy.

On the whole, based on the findings discussed above, Relish appears to be a key regulator of different signaling pathways, including Toll, IMD, and autophagy. Because of the variations in these results, it is possible that there is a -crosstalk between Toll, IMD and autophagy processes. On the other hand, Kranzusch et al. (31) reported the binding of CDNs to STING from the *N. vectensis* and supported the hypothesis that the ancestral biological role of STING in metazoans was to recognize CDNs. Bacteria synthesize different CDNs and cyclic trinucleotides, some of which are capable of activating the *Drosophila* STING gene (66). However, in contrast to Martin et al. (37), who suggested that bacterial c-di-GMP could induce a STING-dependent immune response to *L. monocytogenes* infection. A recent study found no evidence of a contribution of STING or induction of antimicrobial peptides in response bacterial c-di-GMP released after *L. monocytogenes* infection (53). Thus, further studies are required to clarify these discrepancies by considering other parameters such as species variations, insects microbiome, etc.

## Negative-Regulators of STING in Insects

Cytosolic RNA or DNA has been identified as a possible immune inducer that has the potential to activate a robust innate immune response against pathogens for the host defense (67–70). It is the STING that is responsible for the induction of innate immune responses by sensing cytosolic RNA or DNA (32, 71–76). Furthermore, if the STING is not regulated, the persistent activation of immune responses triggered by RNA or DNA could results in uncontrolled innate immune responses that could have an impact on physiological process of the host. Thus, it appears to be essential that there would be a mechanism to keep the effect of persistent activation of STING in a cell under control. In mammals, several molecular mechanisms have been identified that reduce STING activity, such as accelerated degradation by TRIM30α (77), ULK1-mediated phosphorylation (78), impediment of its interaction with TBK1 by NLRC3 (79), RNF5-mediated ubiquitination and degradation (80) and so on. Because of the limited number of studies that has been done on this molecular mechanism of immune responses in insects, only a few STING regulators have been identified in different insect species in recent years.

Roquin is a novel RING-type ubiquitin ligase family member that has recently been discovered in insects. These proteins control the production of T-lymphocytes in vertebrates, and loss of their activity can cause an imbalance of T-lymphocyte (81). The proteins are extremely well conserved, and they have a novel ROQ domain that is resided on the N-terminus of the protein (82). A recent study reported that this protein exists in *Drosophila* and that it is linked with STING regulation (83). The STING regulators in *Drosophila* were identified after screening nine genes encoding the ubiquitin ligase in S2 cells *in vitro* and analyzing their effect on STING-dependent immune responses. The authors reported a negative correlation between roquin protein and STING signaling. In addition, over-expressing of roquin in S2 cells has been shown to inhibit STING-dependent immune response. In contrast, roquin depletion resulted in uncontrolled production of antimicrobial peptides and the reduction of the replication of *L. monocytogenes* (83), suggesting that roquin is a potential negative regulator of STING-dependent innate immune response in insects. It has been shown that the ROQ domain of roquin is flanked by a RING and CCCH finger motif (84). It is still unclear what biological roles the RING-1 zinc finger of roquin performs; however, future studies may shed light on its involvement in the regulation of STING activity. However, it has been demonstrated that proteasome and Polyubiquitylation activity are required for rapid breakdown or degradation of mRNAs that contain destabilizing ARE elements in their 3’-untranslated regions (85). Thus, it appears that roquin may stimulate the ubiquitin-mediated degradation of proteins that control mRNA stability or that ubiquitin-tagging may alter the association or localization of mRNA modulating proteins.

**Figure 4:**
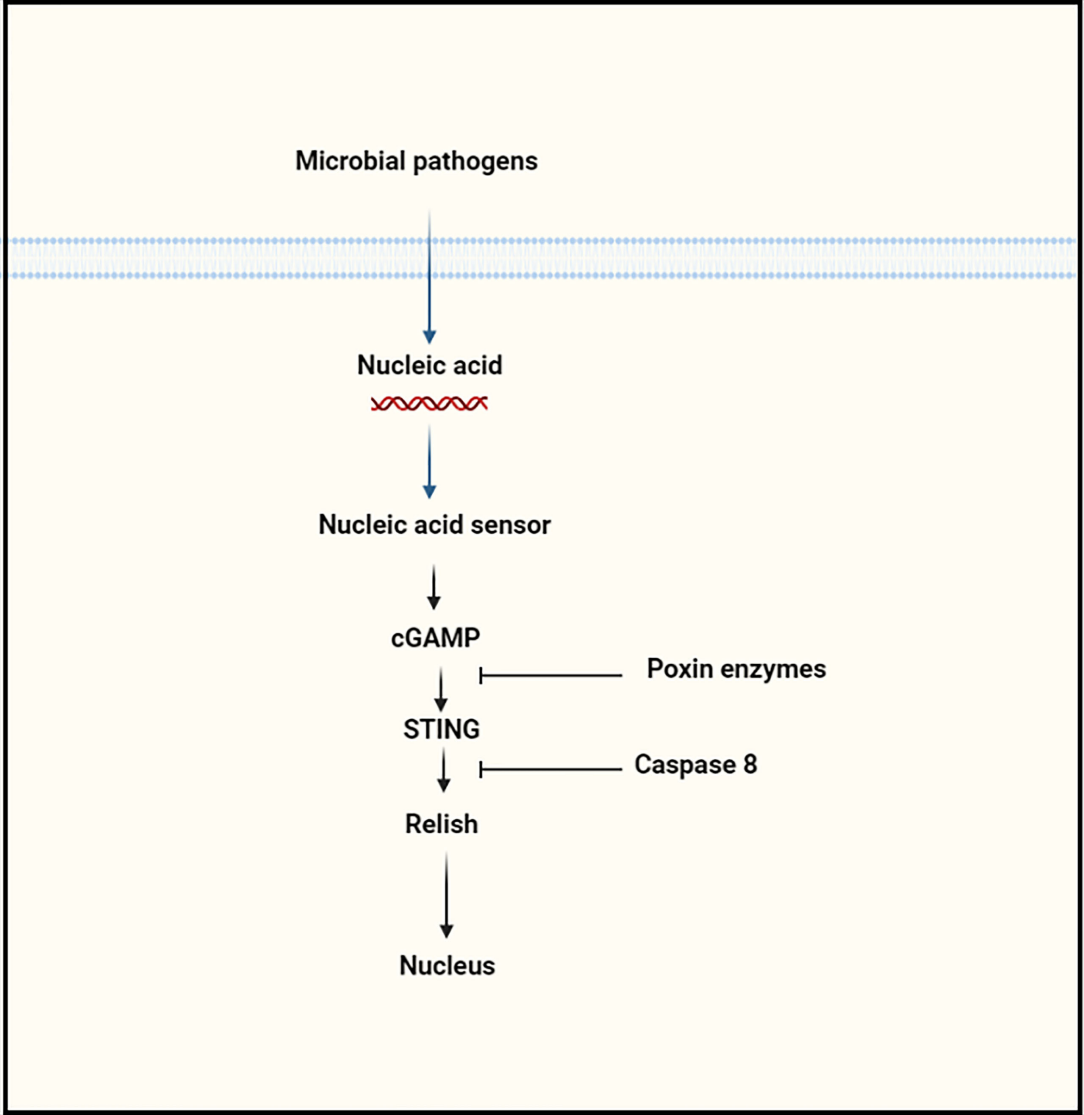
The regulation of cGAS–STING signaling pathway by proteins at various levels.

In addition to the Roquin protein, another study suggested the interaction of Caspase 8L with STING protein based on the *in vivo* and *in vitro* experiments. In comparison to Dredd, the caspase 8L protein shares 70% identity with the N-terminus domain but does not include the C-terminus caspase domain, which is implicated in the cleavage of Relish, implying that while caspase 8L may be involved in Relish processing, it may perform the different biological role (40). In BmE cells, over-expression of Caspase 8L together with Relish has been shown to repress Relish after cGAMP or BmNPV stimulation in the cells. *In vitro*, a caspase 8L deficiency results in a high level of resistance to viral infection, suggesting that caspase 8L is involved in negatively regulating STING-dependent signaling in silkworm, *Bombyx mori*, and possibly in other insect species (40). However, the precise participation of Caspase 8L to the regulation of STING-dependent signaling needs to be determined in more detail in insects (**Figure 4**).

All of these results show that roquin and caspase are involved in the negative regulation of virally induced STING-dependent signaling and innate immunity. Although these studies data demonstrating the involvement of certain proteins in the modulation of STING cascade; nevertheless, the exact molecular mechanisms through these proteins contribute to the negative control of STING-dependent signaling remain still an unanswered questions.

## Innate Immune Evasion Strategies of Pathogens in Insects

In order to complete their life cycle within the host, microbial pathogens may devise strategies to evade the host immune system (86–90). Thus, in order for pathogens to successfully commence replication, they must prevent or inhibit activation of nucleic acid sensors (cGAS or cGAS-like). In mammals, several studies have highlighted the mechanism by which pathogens control the host immunity. For example, DNA viruses (such as retroviruses and herpesviruses) shield DNA within the viral capsid so that it does not become detected by cytosolic nucleic acid sensors until it reaches the nucleus (91–97). The activation of STING by BmNPV (DNA virus) infection was recently described by Hua et al. (40), and it is likely that baculoviruses may also use the same strategy to bypass the host’s immune system.

Another strategy to restrict activation of cGAS or cGAS-like is to target cGAS or cGAS-like for degradation, decreasing levels of this nucleic acid sensor and reducing 2′3′-cGAMP production. This is accomplished in numerous ways by both RNA and DNA viruses. In mammals, poxvirus immune nuclease (poxin) is involved in the degradation of 2′3′-cGAMP and also blocks the stimulation of anti-viral immune signaling through the STING signaling cascade during infection of poxvirus. The poxin of most poxviruses occurs as a fusion to a C-terminal domain with homology to mammalian schlafens (5, 98, 99). The enzymes also have also been reported in insects, and it seems to have a prominent function for 2′3′-cGAMP signaling in insects. Several studies separately documented the STING signaling pathway in insects that drives NF-kB and autophagy signaling to prevent viral infection (40, 41, 46). Recent work on insects involves a cGAS-like molecule to activate STING signaling (43, 44, 100). It seems that poxin enzymes of insect viruses likely prevent activation of immune response comparable to the biological role of poxin of mammalian poxviruses.

In summary, since our understanding is increasing regarding cGAS–STING signaling insects, nevertheless bacterial and viral components that may target this signaling pathway for the successful replication need to be elucidated. So far, our knowledge is rather preliminary regarding the negative or positive regulation of this pathway. Therefore, future studies are required to determine how microbial pathogen suppresses the STING immune signaling activation in insects. In addition, how insect viruses poxin sense the cGAS-like molecules at a molecular level and how microbial pathogen escape, shape the structure and modulation of STING-dependent immune pathways in different insect species.

## Future Directions

The biological roles of the cGAS-STNG signaling pathway in the immune system of mammals have been well-established. In contrast, this pathway has recently been discovered in insects, and biochemical evidences supports the immunological functions of the cGAS-STING pathway in insects. The lack of extensive literature is limiting for any direct and clear conclusions. But, with this research background, we endeavored to review available knowledge and develop preliminary conclusions based on our understanding of the subject. Furthermore, research on insects cGAS-STING is still in its early stages, and more investigations are needed before a definite conclusion can be drawn on this topic.

## Author Contributions

The authors’ responsibilities were as follows: LD, MNA, and BT designed this review article. QL, SK, YT, and WH downloaded material and write down draft. QL, SK, and MNA draw the diagram and proofread the article. All authors contributed to the article and approved the submitted version.

## Funding

This work was funded by the Natural Science Research General Program of Jiangsu Provincial Higher Education Institutions (21KJA240003), the Natural Science Foundation of Jiangsu Province (BE2020673), the Doctorial Start-up Fund of Southwest University (SWU020023), the National Key R&D Program of China (2019YFD0900404-05), the Industry-University-Research Cooperation Project of Jiangsu Province (BY2020644), the Open Funding Project of Anhui Province Key Laboratory of Aquaculture & Stock Enhancement (AHSC202001), the 16th Six Talents Peak Project of Jiangsu Province (NY-126), the National Natural Science Foundation of China (32070526), the Jiangsu Agriculture Science and Technology Innovation Fund (CX(18)3027). The study was sponsored by the Qing Lan Project of Jiangsu Province, and the “Outstanding Young Talents” of YCTU.

## Conflict of Interest

The authors declare that the research was conducted in the absence of any commercial or financial relationships that could be construed as a potential conflict of interest.

## Publisher’s Note

All claims expressed in this article are solely those of the authors and do not necessarily represent those of their affiliated organizations, or those of the publisher, the editors and the reviewers. Any product that may be evaluated in this article, or claim that may be made by its manufacturer, is not guaranteed or endorsed by the publisher.
